# Effectiveness of Small Bites Closure in Reducing Incisional Hernia at Specimen Extraction Sites Following Minimally Invasive Colorectal Cancer Surgery

**DOI:** 10.7759/cureus.94815

**Published:** 2025-10-17

**Authors:** Damien Mony, George Ninkovic-Hall, Conor Magee, Jeremy Wilson

**Affiliations:** 1 Department of General Surgery, Wirral University Teaching Hospital NHS Foundation Trust, Wirral, GBR; 2 Department of Vascular Surgery, Royal Liverpool University Hospitals Trust, Liverpool, GBR

**Keywords:** abdominal incisional hernia, colorectal cancer, colorectal cancer surgery, extraction site, small bites vs. mass closure

## Abstract

Introduction and aims: This study compares the incidence of incisional hernia (IH) at extraction sites in minimally invasive colorectal cancer resections using the small bites closure technique versus mass closure.

Methods: We conducted a single-centre retrospective study at our UK hospital, including all adult patients who underwent laparoscopic or robotic colorectal cancer resections between 2018 and 2023. Patients were divided into two groups based on the closure technique: small bites and mass closure. The incidence of IH at the extraction site was confirmed either clinically or radiologically during routine follow-ups. Fisher's exact test was used to compare IH rates between the groups, with significance set at p<0.05.

Results: A total of 246 patients were included in the study, divided into small bites (n=112) and mass closure (n=134) groups, with a median age of 69 years (range 31-89). The overall incidence of IH was 13.4% (n=33). IH rates were significantly lower in the small bites group (5.3%; n=6) compared to the mass closure group (19.9%; n=27) (p=0.006). Midline extraction sites had a higher IH incidence (16.8%) compared to off-midline sites (1.8%) (p=0.003). Additionally, patients with a BMI ≥30 kg/m² had a higher IH incidence (24.2%) compared to those with a BMI <30 kg/m² (8.9%) (p=0.003).

Conclusion*: *This study suggests that the small bites technique significantly reduces the incidence of IH at extraction sites in minimally invasive colorectal cancer resections compared to mass closure. Patient factors such as body mass index (BMI) and extraction site location are also critical in IH development.

## Introduction

Incisional hernia (IH) is a well-recognised complication following abdominal surgery and is associated with significant morbidity and institutional costs [1]. The estimated incidence of IH ranges from 5% to 20% in both open and minimally invasive procedures [2]. The risk surrounding IH lies with patient symptomology and complications, including incarceration, strangulation, and obstruction [3]. Repair of IHs can be complex and may have high long-term recurrence rates, highlighting the need for preventative measures [4,5]. Given the increasing proportion of colorectal cancer resections being performed laparoscopically and robotically, attention should be given to improving rates of IH in this cohort [6-8].

Several patient factors contribute to the development of IH, including body mass index (BMI), smoking status, diabetes, pregnancy, medications, and collagen disorders. Unlike the choice of closure technique, these risk factors fall outside the surgeon's control when it comes to reducing the incidence of IH.

The efficacy of the small bites technique compared to mass closure in midline laparotomy to reduce rates of IH has been shown to be effective [9-13]. This has not been demonstrated for the specimen extraction site of minimally invasive colorectal resections.

In 2018, our institution introduced a standardised closure technique with the aim of reducing IH, which incorporated the small bites technique using 2-0 polydioxanone (PDS) sutures. The use of this technique at the extraction site of cancer resections was not mandatory and was utilised based on the surgeon's preference.

The aim of this study was to compare local rates of IH at extraction sites of laparoscopic and robotic colorectal cancer resections following the small bites technique versus mass closure.

## Materials and methods

Study design and patients

We undertook a single-centre, retrospective observational study at Arrowe Park Hospital, Wirral, UK. Data were analysed from all adult patients at our institution who underwent laparoscopic or robotic colorectal cancer resections for a five-year period (2018-2022). 

The small bites technique was introduced to the colorectal unit at our trust as part of a standardised abdominal wall closure pathway in 2018. This was following a review of the surrounding evidence, and practitioners were given formal training on this technique using 2-0 PDS suture. 

Data for eligible patients were extracted from our local colorectal cancer remote-surveillance database. The clinical notes, operative notes, postoperative clinic letters, and radiological scans were then reviewed for the exposure and outcomes. Details extracted included the following: patient age, sex, BMI, smoking status, diabetes, cancer location, cancer staging, type of resection performed, closure technique, suture material used, and location of extraction site. The length of the extraction site incisions was typically 5 cm or less. The smoking status of the patient was confirmed as positive if they had smoked within the preceding 12 months of the operation. 

**Figure 1:**
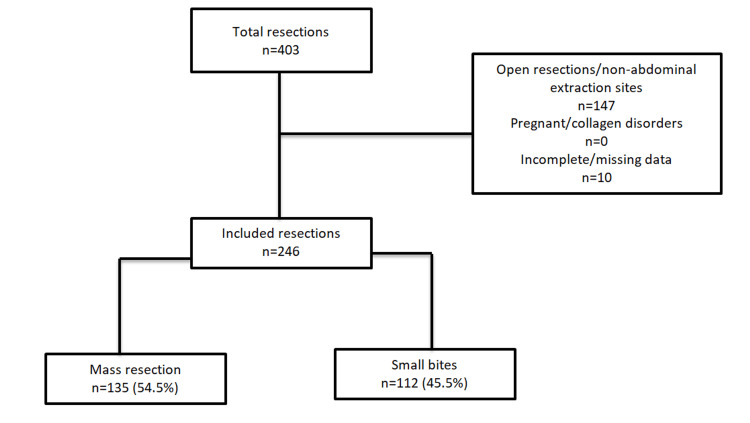
Patient screening and closure cohorts

Figure 1 depicts the patient screening and closure cohorts.

Inclusion criteria 

All laparoscopic or robotic colorectal cancer resections in adult patients 18 years of age and above were screened. Only IHs at the specimen extraction site were considered in this study. Any port site, parastomal, or non-IHs were not included in the analysis. 

Exclusion criteria

Open, laparoscopic, or robotic converted to open and minimally invasive resections with non-abdominal extraction sites were removed from this study. Pregnant patients and those with collagen disorders or re-operation within the same hospital admission were also excluded from this study. Any patients with incomplete or missing data with respect to the closure technique in the operation note were excluded. 

Outcome measures 

Extraction site IH following resection was confirmed either clinically by the operating surgeon in the outpatient clinic or radiologically reported via yearly remote-surveillance computed tomography (CT) scans of the thorax, abdomen, and pelvis. All patients were automatically entered into our local cancer remote-surveillance programme which involved the following postoperative protocol: annual CT scan, carcinoembryonic antigen (CEA) tumour marker blood test six-monthly for two years and then yearly thereafter, and colonoscopy at one and four years. 

All patients were reviewed within eight weeks following hospital discharge and underwent clinical examination by the operating surgeon. All patients had received a minimum of one year of clinical or radiological follow-up at the time of data analysis. 

The rates of IH at the extraction site between the mass closure and small bites groups were then compared. 

Statistical analysis 

Data was assessed using IBM SPSS Statistics for Windows, Version 29.0 (IBM Corp., Armonk, New York, United States), with a p-value of less than <0.05 considered statistically significant using Fisher's exact test.

Further subgroup analysis of pre-defined covariates in the two stitch groups was conducted, including BMI, diabetes, smoking status, and location of incision.

## Results

A total of 246 patients were included in the study from February 2018 and January 2023. Mass closure and small bites were utilised in 134 (54.5%) and 112 (45.5%) cases, respectively. In our data, 117 (48%) participants were female and 129 (52%) were male, with a median age of 69 years (range 31-89). Of our patient cohort, 23 (9%) were smokers, 32 (13%) were diabetic, 11 (4%) developed extraction site wound infections, and there was a mean BMI of 27.5 kg/m² (range 17.0-45.0 kg/m²; SD=5.1). The BMI was categorised into above and below 30 kg/m² for analysis based on the World Health Organization (WHO) obesity definition [14]. 

Of all resections, 136 (54.6%) were performed for T3 cancers, with 172 (69.1%) classified as N0. Regarding resection technique, 195 (79.3%) cases were laparoscopic and 51 (20.7%) were performed robotically. 

There were comparable patient demographics across both groups. However, there was a higher proportion of patients in the small bites group with off-midline extraction sites compared to the mass closure group (29.5% vs. 17.2%). Table 1 compares the baseline characteristics and operative variables between the two stitch techniques. 

**Table 1:** Patient demographics and operative variables in each stitch group

Variable		Mass closure		Small bites		Total	
		n	%	n	%	n	%
Sex	Male	65	48.5	64	57.1	129	52.4
	Female	69	51.5	48	42.9	117	47.6
Age (years)	≤50	9	6.7	8	7.1	17	6.9
	51-70	70	52.2	47	42	117	47.6
	70+	56	41.8	57	50.9	139	56.5
Smoking status	Smoker	13	9.7	10	8.9	23	9.3
	Non-smoker	121	90.3	102	91.1	223	90.7
BMI (kg/m^2^)	≥30	38	28.4	28	25	66	26.8
	<30	96	71.6	84	75	180	73.2
Diabetes	Diabetic	19	14.2	13	11.6	32	13
	Non-diabetic	115	85.8	99	88.4	214	87
Procedure type	Laparoscopic colonic resection	65	48.5	41	36.6	106	43.1
	Laparoscopic anterior resection	43	32.1	41	36.6	84	34.1
	Laparoscopic abdomino-perineal resection	4	3	0	0	4	1.6
	Robotic abdomino-perineal resection	0	0	1	0.9	1	0.4
	Robotic anterior resection	21	15.7	29	25.9	50	20.3
	Robotic colonic resection	1	0.7	0	0	1	0.4
Extraction site	Midline	111	82.8	79	70.5	190	77.2
	Off-midline	23	17.2	33	29.5	56	22.8

Outcomes 

All 246 (100%) had both clinical and radiological follow-up at one year post-operation. With respect to yearly radiological CT follow-up, 168 (68.3%) patients had repeat scans at two years, 111 (45.1%) at three years, and 54 (22%) at four years. For clinical follow-up, 59 (24%) patients were seen at two years, 20 (8.1%) at three years, and 18 (7.3%) at four years post-operation. 

During the five-year observation period, 33 (13.4%) patients developed IH through the specimen extraction site across both groups. In the mass closure group, 27 (19.9%) patients developed IH, compared to six (5.3%) in the small bites group (p=0.006). 

Across both groups, 32 (16.8%) patients developed IH when a midline extraction site was used. Conversely, one (1.8%) patient had confirmed IH with an off-midline incision. This difference was statistically significant (p=0.003). When comparing IH incidence with BMI, 66 patients had a BMI greater than or equal to 30 kg/m², of which 16 (24.2%) developed IH. This is compared to 16 (8.9%) confirmed IHs in the 180 patients with a BMI less than 30 kg/m² (p=0.003). We found no statistically significant relationship between IH and diabetes (p=1.00) and smoking (p=0.220) in the total population.

Most hernias were detected solely radiologically in 21 (63.6%) patients, followed by both clinically and radiologically in nine (27.3%) patients and solely clinically in three (9.1%) patients. 

Small bites versus mass closure

The median ages were 71 and 67 years of age in the small bites and mass closure groups, respectively. The mean BMI in the small bites group was 27.1 kg/m² and 27.4 kg/m² in the mass closure group. Further analysis of IH incidence was performed for each stitch group, accounting for patient factors and extraction site, as demonstrated by Tables 2-3. 

**Table 2:** Patient demographics and hernia incidence in the mass closure cohort P-values have been calculated using Fisher's exact test, with values <0.05 considered to be significant.

Variable		Hernia (n=27)	No hernia (n=109)	P-value (Fisher's exact test)
Extraction site	Midline	26	87	p=0.045
	Off-midline	1	22	
BMI (kg/m^2^)	BMI <30	12	84	p=0.002
	BMI ≥30	15	25	
Smoking status	Smoker	1	13	p=0.311
	Non-smoker	26	96	
Diabetes	Yes	4	15	p=1.000
	No	23	92	

**Table 3:** Patient demographics and hernia incidence in the small bites cohort P-values have been calculated using Fisher's exact test, with values <0.05 considered to be significant.

Variable		Hernia (n=6)	No hernia (n=106)	P-value (Fisher's exact test)
Extraction site	Midline	6	73	p=0.178
	Off-midline	0	33	
BMI (kg/m^2^)	BMI <30	5	79	p=1.000
	BMI ≥30	1	28	
Smoking status	Smoker	0	10	p=1.000
	Non-smoker	6	96	
Diabetes	Yes	0	13	p=1.000
	No	6	93	

Table 4 compares the time at which IHs are detected in both closure technique groups. 

**Table 4:** Time at hernia detection

Year postoperatively	Small bites		Mass closure	
	n	%	n	%
≤1 year	4	66.7	11	40.7
≤2 years	1	16.7	12	44.4
≤3 years	1	16.7	3	11.1
>3 years	0	0	1	3.7

## Discussion

The small bites technique was first described in 2009 by Millbourn et al. which utilised a self-locking knot in conjunction with small tissue bites (approximately 5 mm) and finished with an Aberdeen knot to achieve a suture length-to-wound length ratio of greater than 4 [9]. This is in contrast to mass closure which is a continuous running stitch with larger tissue bites of approximately 10 mm incorporating all layers of the abdominal wall except the skin and subcutaneous fat [10,11]. Millbourn et al. found reduced rates of IH when the small bites technique was utilised in comparison to large bites [9]. Following this, the STITCH trial and subsequent studies mirrored Millbourn and colleagues' findings of reduced IH incidence with small bites [10-13]. It is suggested that this technique will primarily involve aponeurosis during closure, therefore minimising the risk of necrosis of surrounding muscle and adipose tissue which optimises wound healing and integrity [10]. Furthermore, experimental data have suggested a more equal tension distribution across the wound with this technique when compared to large bites [15]. 

Our data suggests a statistically significant reduction in IH incidence in the small bites closure cohort compared to mass closure. This is in keeping with existing literature which highlighted these findings in patients undergoing laparotomy. However, there is limited data on the implications of this technique at the specimen extraction site of minimally invasive colorectal cancer resections.

The patient demographics in both stitch groups were comparable with similar proportions of diabetic patients, smokers, and BMI. There was a higher percentage of patients with off-midline extraction sites in the small bites cohort; however, there was no statistically significant relationship between IH incidence and incision site in this group. Therefore, the reduced IH occurrence in this group may not be attributable to the increased proportion of patients with off-midline extraction sites. When accounting for patient factors in this stitch group, there was no statistically significant patient variable with respect to IH incidence. In the mass closure group, incision site and BMI both had a statistically significant relationship with IH development. This may have contributed to the increased IH incidence in this stitch group, rather than the closure technique alone. 

Grantcharov and Rosenberg's meta-analysis on the impact of incision site for the development of IH underscored the reduced incidence of IH when an off-midline, transverse incision is utilised [16]. This is proposed to be due to the greater disruption of transversely orientated abdominal wall fibres and increased lateral wound tension when a midline incision is taken [16-18]. Our data also supports this with increased IH rates with midline incisions in the overall cohort and mass closure group. The greater incidence of IH in the mass closure cohort may have been affected by the increased proportion of resections featuring a midline extraction site. 

Our data also reinforces the well-understood relationship between BMI and IH. In the total population and mass closure group, BMI was a statistically significant covariate in IH development [18]. 

Over half the IHs in both stitch groups were detected exclusively through radiological modalities. Only a minority of hernias were confirmed through clinical examination alone. This finding is supported by the STITCH trial which found greater IH detection when radiological imaging was utilised [10]. This could be accounted for in this study by our remote-surveillance programme which precludes the need for attendance in the outpatient clinic for history and examination as a mode for surveillance. Additionally, the period of this study included the COVID-19 pandemic which limited face-to-face clinic appointments. Although not the aim of this study, we have no data on patient-reported outcome measures to ascertain the individual burden of IH with respect to symptoms. Therefore, it is difficult to determine the real-world implications of radiologically detected IH on patient symptoms in this cohort. 

The exact timing of IH detection postoperatively varies, especially in the asymptomatic patient. Studies have reported up to 68% of IHs detected within one year post-operation and 80% within three years [19]. Under half of IHs were detected within one year post-operation for both stitch groups. However, 32 (96.9%) of IHs were detected within three years post-operation. Our data revealed that two-thirds of IHs were identified within one year postoperatively in the small bites group. In comparison, there was a higher proportion of IHs detected between one and two years postoperatively in the mass closure group. This could suggest an earlier occurrence of IH when the small bites technique is used compared to mass closure.

Limitations

This study has several limitations. The choice of closure technique was left to the operating surgeon's discretion, as the implementation of small bites closure was not mandatory. Additionally, the suture (0, 1, or 2 PDS) material for the mass closure technique varied by surgeon preference.

Being a retrospective study, there was no randomisation of the stitch groups, which may have introduced bias in the selection of closure techniques. The study also lacked data on the size of the hernial defect, patient-reported outcomes, and precise measurements of the extraction site incision. Moreover, follow-up durations were not consistent across all patients. Other variables which were not examined, such as neoadjuvant chemotherapy, steroid use, and emergency versus elective surgery, may have implications for IH development in this cohort. 

To better understand the relationship between the small bites technique and IH in minimally invasive colorectal resections, prospective, multi-centre, and randomised studies are needed.

## Conclusions

This retrospective study suggests that the small bites technique significantly reduces the incidence of IH at extraction sites in minimally invasive colorectal cancer resections compared to mass closure. This is in line with existing literature comparing these two techniques in patients undergoing midline laparotomy. Given the high proportions of colorectal cancer resections performed with a minimally invasive approach, adoption of this technique may reduce IH burden and its associated complications in this population. However, it is important to note that patient and operative factors, such as raised BMI and location of the extraction site, certainly play a crucial role in the development of IH. Furthermore, this is a small retrospective study with the usual inherent limitations, but there is a strong signal regarding the potential benefit of small bites closure that warrants further investigation with a larger randomised study.
